# Enforcing trustworthy cloud SLA with witnesses: A game theory–based model using smart contracts

**DOI:** 10.1002/cpe.5511

**Published:** 2019-09-01

**Authors:** Huan Zhou, Xue Ouyang, Jinshu Su, Cees de Laat, Zhiming Zhao

**Affiliations:** ^1^ Informatics Institute University of Amsterdam Amsterdam The Netherlands; ^2^ School of Computer Science National University of Defense Technology Changsha China; ^3^ School of Electronic Sciences National University of Defense Technology Changsha China

**Keywords:** blockchain, cloud computing, game theory, service level agreement, smart contract

## Abstract

There lacks trust between the cloud customer and provider to enforce traditional cloud SLA (Service Level Agreement) where the blockchain technique seems a promising solution. However, current explorations still face challenges to prove that the off‐chain SLO (Service Level Objective) violations really happen before recorded into the on‐chain transactions. In this paper, a witness model is proposed implemented with smart contracts to solve this trust issue. The introduced role, “Witness”, gains rewards as an incentive for performing the SLO violation report, and the payoff function is carefully designed in a way that the witness has to tell the truth, for maximizing the rewards. This fact that the witness has to be honest is analyzed and proved using the Nash Equilibrium principle of game theory. For ensuring the chosen witnesses are random and independent, an unbiased selection algorithm is proposed to avoid possible collusions. An auditing mechanism is also introduced to detect potential malicious witnesses. Specifically, we define three types of malicious behaviors and propose quantitative indicators to audit and detect these behaviors. Moreover, experimental studies based on Ethereum blockchain demonstrate the proposed model is feasible, and indicate that the performance, ie, transaction fee, of each interface follows the design expectations.

## 1 INTRODUCTION

Cloud computing is a popular business model nowadays for sharing physical resources among multiple tenants. A cloud customer can rent and use various resources as service, including computing, storage, and network, from the provider through a network (typically via the Internet) without maintaining the physical hardware. Although convenience is conveyed, this causes the challenge of “*Cloud Performance Unpredictability*”^1^ when migrating time‐critical applications onto clouds.^2^ Cloud SLA (Service Level Agreement) is therefore proposed to ensure that certain service quality can be met, and in the case of violation, the customer can get the corresponding compensation from the provider. It compensates the customer's loss due to the cloud performance uncertainty from the economic perspective.

Traditionally, SLA is a business concept which defines the contractual financial agreements between the roles who are engaging in the business activity.^3^ As to cloud computing, it is an agreement between the cloud customer and provider on the quality of the cloud service. For instance, the IaaS (Infrastructure‐as‐a‐Service) provider, Amazon Elastic Compute Cloud (EC2), claims that the availability of its data center is no less than 99%. If this number is not achieved, it will pay 30% credits back to the customer as compensation. * However, it is hard to enforce this agreement in practice. The significant challenges that hinder the conceptual SLA to be widely applied in the real‐life industry are (1) there lacks an automatic mechanism to enforce the agreement, especially automate the compensation after SLO violation. The current process involves a lot of manual work such as doing the verification through emails before compensation, ie, the customer needs to submit a claim to EC2 website for its SLO violation^∗^; (2) the provider has more rights in current agreement model, because it has the right to verify the violation and decide whether to compensate the customer. On the contrary, the customer has little space to negotiate about the price or the amount of compensation; (3) the agreement is only between the cloud customer and the provider. It is hard for the customer to prove and convince the provider that the violation has really happened. For example, EC2 requires the customer to provide detailed logs that record the dates and times of each unavailability incident.*

The blockchain^4^ technology brings in a new hint for possible solutions to address these challenges. Especially, the smart contract in Ethereum^5^ makes it possible to automate the SLA on the blockchain. For instance, Nakashima and Aoyama^6^ designed a set of web APIs based on Ethereum to automate the SLA enforcement. They introduce a new role called “*Service Performance Monitor*” into the scenario, which detects the SLO violation and sends the notification. However, this work does not discuss the trust issue for this newly proposed role, which is very important in blockchain‐based systems.

It is challenging to achieve consensus on an event that happens outside of the blockchain. The bridge between the events that on and outside of the chain is called “Oracle”. † One of the solutions is to retrieve data from “oraclize”, ‡ a third trusted company performing as a trustful data source. Town Crier^7^ and TLS‐N^8^ perform as oracles from the hardware and infrastructure perspective, which require specific hardware support. Moreover, these solutions are centralized, which suffer from single‐point failure and are easy to be compromised.

In this paper, a model based on smart contracts is proposed to tackle the challenge of detecting SLO violations in a trustworthy way. A new role termed as “Witness” is added in the traditional cloud service delivering scenario to perform as the service monitor. The witness is designed as an anonymous participant in the system, who desires to gain rewards through offering the violation reporting service. The payoff function for different actions in our agreement model is carefully designed in a way that the witness will have to always behave honestly in order to gain the maximum profit for himself. In other words, the trust issue of the witness in our model is proved by game theory through the use of the Nash Equilibrium principle.

In addition, an unbiased random selection algorithm is developed in our witness model to select a certain number of witnesses for constructing a committee. The committee members are randomly selected, and any entity cannot dominate the selection results, which is essential to avoid the situation that the majority of the delegates are representing the same side, either the customer or the provider. Besides, this algorithm is also capable of eliminating the opportunity of collusion because the committee members are not pre‐determined, and there is little possibility for them to know each other in advance.

Finally, a prototype system §using smart contracts of the Ethereum^5^ blockchain is implemented to automate the SLA lifecycle and empower the fairness between roles, especially for the customer. Rinkeby, ¶ which is a world‐wide blockchain test net for developers to debug the developed smart contracts, is leveraged in this paper to conduct the experiments. The experimental study demonstrates the feasibility of our witness model and system performance.

The rest of this paper is organized as follows. Section 2 discusses the related work on cloud SLA and blockchain. Then, Section 3 describes the witness model design with smart contracts. In Section 4, we detail crucial techniques including the unbiased random selection algorithm and the payoff function design, the trustworthiness of which is proved by Nash Equilibrium principle; Section 5 introduces the prototype implementation and experiments; Section 6 summarizes this paper with conclusion and future work.

## 2 RELATED WORK

SLA is a hot research topic, specifically well discussed in the context of cloud computing. It establishes the quality of service agreement between the service provider and the customer, which ensures the customer's benefit even when the resource planning^9^, ^10^ has failed to satisfy the application's requirements due to the cloud failures. This agreement is especially important for operating time critical applications on clouds.^11^, ^12^, ^13^ A typical SLA lifecycle consists of different enforcement phases, including negotiation, establishment, monitoring, violation reporting, and termination.^3^ Most of the research work focuses on three aspects, ie, (1) syntax definition of the SLA terms and parameters. Its goal is to standardize the representation and make SLA easy to be processed online by computer systems. There is a set of domain specific languages proposed to solve the issue, such as SLAC^14^ and CSLA^15^; (2) resource allocation techniques to ensure the SLA. The work in this aspect focuses on the algorithm to optimize resource allocation, making sure the SLO violation does not happen. Here, SLA is considered as constraints^16^; (3) systems or methods to address the issue in some specific phase of the SLA lifecycle. In this aspect, a systematic survey^3^ on cloud SLA concludes that papers they found are distributed to SLA phases as follows: negotiation and establishment(22%), monitoring and deployment(73%), SLO violation management (3%), and reporting (1%). Here, the goal of negotiation is to maximize either the provider or the customer's rewards by adopting some negotiation strategy.^17^ For example, Groléat and Pouyllau^18^ leveraged reinforcement learning method to learn and update the negotiation policy. The monitoring phase is mainly about what to monitor and how to automate the process.^19^ However, the most challengeable phase, violation management and report, is seldom touched. In industry, Amazon CloudWatch service #is an example that the provider automates monitoring and notification. In this case, the provider has to be trusted by the customer. Müller et al^20^ developed a platform, named as SALMonADA, to deal with the SLO violation at runtime. It works as a third trusted party to perform the monitoring and violation reporting. All these work treat the violation reporting is trustworthy as an assumption. Nevertheless, it is precisely the trust issue of cloud systems,^21^ making it difficult to let the provider and customer have consensus on the violation in reality.

Smart contract concept was first proposed by Nick Szabo in 1994, which digitally facilitates, verifies, and enforces a contract through a computer protocol.^22^ Scoca et al^23^ combined this concept with cloud SLA negotiation. It focuses on the semantics expressions of smart contracts to automate the negotiation. However, it lacks a trustworthy underlying platform to execute the smart contract. Hence, the smart contract requires a strong assumption that no one can tamper its execution. Town Crier^7^ and TLS‐N^8^ ensure the trustworthy execution and communication environment from hardware and transmission protocol, respectively. However, they are either centralized or require specific hardware support.

Blockchain^4^ is a promising technique to be the execution platform since the interactions on the chain are immutable. Especially, Ethereum^5^ first realizes to execute a general‐purpose program on its blockchain. They design several programming languages, which make it possible to implement smart contracts. Nakashima and Aoyama^6^ leveraged Ethereum and designed a set of web APIs. They attempt to automate the SLA lifecycle enforcement on the blockchain. A new role called “*Service Performance Monitor*” is introduced in their paper, which is responsible for the violation management and reporting. However, whether the violation reports sent to the blockchain can be trusted is not discussed. In essence, this is still a gap for blockchain based systems. That is how to credibly record a random event happening outside of the blockchain onto the chain. It is called “*oracle*”,^†^ which is a party performing as a “data‐carrier” for the blockchain. Oraclize^‡^ is a third trusted company currently offering the service as an oracle. Nevertheless, a third trusted party exists single‐point failure and deviates from the decentralization idea of blockchain. Hence, ChainLink^24^ works on distributed oracles. Only when an agreement is achieved among the oracles, the result data of the event can be carried onto the chain or trigger a transaction. However, it has some downsides, such as no incentive for individuals to do this, requiring individuals being independent and trustworthy, the consensus issue among oracles, etc.

## 3 THE WITNESS MODEL USING SMART CONTRACT

In this section, the related roles in the proposed model design are introduced, especially the witness role. Then, the overall system architecture for SLA enforcement with smart contracts on a public blockchain is illustrated, followed by a detailed description of the major responsibility of witnesses, ie, SLO violation detection and reporting.

### 3.1 The witness Role and assumptions

There are mainly two roles in the traditional cloud SLA lifecycle. One is the cloud provider role, *P*, which offers cloud service. The other is the cloud customer role, *C*, which consumes the cloud service and pay the service fee. To demonstrate the critical contribution of our work, we take a basic example to formulate our problem as follows.

A cloud provider, *p*, is an IaaS (Infrastructure‐as‐a‐Service) provider. It provisions VMs (Virtual Machine) on demand with public addresses for its customers to use. For instance, according to the request of a customer *c*, provider *p* provisions a VM with a public IP address, *IP*_*pub*_. During the service time, *T*_*service*_, the customer, only the customer *c* is able to SSH and login to the VM through the corresponding address *IP*_*pub*_. In this case, the SLA can be that the provider *p* claims that during the service time, the provisioned VM will always be accessible. If this is true, the customer *c* must pay the service fee, *F*_*service*_, to the provider *p* after the ending of the service. Otherwise, the customer *c* can acquire a compensation fee, *F*_*compensation*_. That is, the customer *c* only needs to pay *F*_*service*_−*F*_*compensation*_ to the provider *p* in the end, where we assume that *F*_*service*_>*F*_*compensation*_. For the latter case, if it happens, we define it as an SLO violation event. Besides, it is worth to mention that we should exclude the case that the inaccessibility is caused by the customer's own network problem, to be a violation event.

With only these two roles in the agreement, it is hard to ensure that the provider can get paid or the customer can get compensation paid back if the service fee is prepaid. Hence, we leverage blockchain to play as the trusted party to afford a platform for these two roles and enforce these monetary transmissions. However, it is still extremely difficult to convince both roles whether the violation happens and whether it is caused by the customer's own network problem. We therefore bring in another new role in the traditional SLA lifecycle, named as witness role, *W*. They are also the normal participants in the blockchain and volunteers to take part in our SLA system to gain their own rewards through offering monitoring service. In order to solve the trust issue, a set of *N* witnesses, {*w*_1_,*w*_2_,…,*w*_*N*_}, is selected to form a committee in a specific SLA lifecycle. They together report the violation event and may obtain witness fee, *F*_*witness*_, as rewards from both the provider and the customer. Moreover, the wallet address of a specific role on the blockchain is denoted as a filed value of it, *x*.*address*. For instance, *w*_*k*_.*address* is the wallet address of witness *w*_*k*_.

In this paper, we make the basic assumption on the witness role that it is always selfish and aims at maximizing its own rewards.

### 3.2 Overall system architecture

**FIGURE 1:**
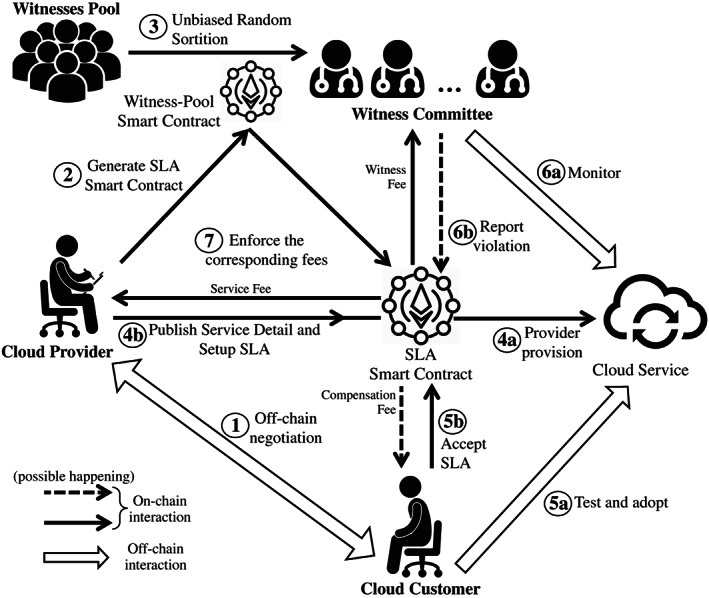
System architecture for cloud SLA enforcement

Figure 1 illustrates the system architecture we design for cloud SLA enforcement. It consists of two types of smart contracts on the blockchain. One is the witness‐pool smart contract, which is the fundamental smart contract of the system to manage all the registered witnesses. The other type is the SLA smart contract for a specific SLA enforcement. The responsibilities of the witness‐pool smart contract include witness management, specific SLA contracts generation, and witness committee construction. Any user of the blockchain, who has a wallet address, can register its wallet address into the witness pool to be a member of witnesses. They can keep themselves online and wait to be selected for some specific SLA contract. The incentive for the witness to participant in this system is to obtain rewards. Moreover, the more witness participants in the system, the more reliable and trustful the system would be.

The entire SLA lifecycle then becomes as follows. Certain provider *p* first leverages the smart contract of witness pool to generate an SLA smart contract for itself. Prior to setting up SLA, the customer *c* should negotiate with the provider *p* about the detailed SLA terms, including *T*_*service*_, *F*_*service*_, *F*_*compensation*_, etc. Here, one of the most important terms is to determine *N*, which is the number of witnesses would be involved in the enforcement of this SLA. The more witnesses involved in an SLA, the more credible the violation detection results are. On the other hand, however, the more witness fee would be paid, and both the customer and the provider need to afford this fee equally. According to this negotiation, the provider can reset these parameters of the generated SLA contract. Afterward, a set of *N* witness members can be selected to form a witness committee through the selection algorithm in Section 4.1, which is implemented by the witness pool smart contract on the blockchain. We design the algorithm to be random and being able to convince both, *c* and *p*, that most ones in the witness committee are independent and would not belong to the adverse role. After dynamically selecting the witness committee member, only the candidate witness can join the specific SLA contract. Meanwhile, the provider provisions its cloud service for the customer to use and is able to publish its service details in the SLA contract. The witnesses from the committee therefore start to monitor the service. In the case of our problem assumption in Section 3.1, the provider *p* provisions a VM on demand and notify the public address *IP*_*pub*_ to all the committee members and customer *c* through the service detail field of the SLA contract. Therefore, the customer can use the VM, and each witness starts to “ping” the address *IP*_*pub*_ constantly. If the violation happens during the service time, ie, the address *IP*_*pub*_ is not accessible, the witness can report this event immediately. However, this is a naive example. In real SLAs of more complex scenarios, the service monitoring component can be negotiated and provided by the provider and customer. Besides, this component can be delivered in the form of containers, which is lightweight and portable. Then, the witness is able to first download the container and query the container to detect SLO violation. Section 3.3 describes how the violation state can be finally determined through these witnesses' reports. If the violation event is approved, then the customer can get back its compensation fee. All the dash lines in Figure 1 mean it may happen according to the actual event. Anyhow, the provider and witnesses from the committee are able to get corresponding fees.

### 3.3 SLO violation detection and report

The critical role in our SLA enforcement system is the witness. It takes the responsibility of monitoring the service and determining whether there is a violation. In this section, we present our smart contract model in a specific SLA lifecycle to demonstrate crucial functionalities of a witness, ie, SLO violation detection and report.

**FIGURE 2:**
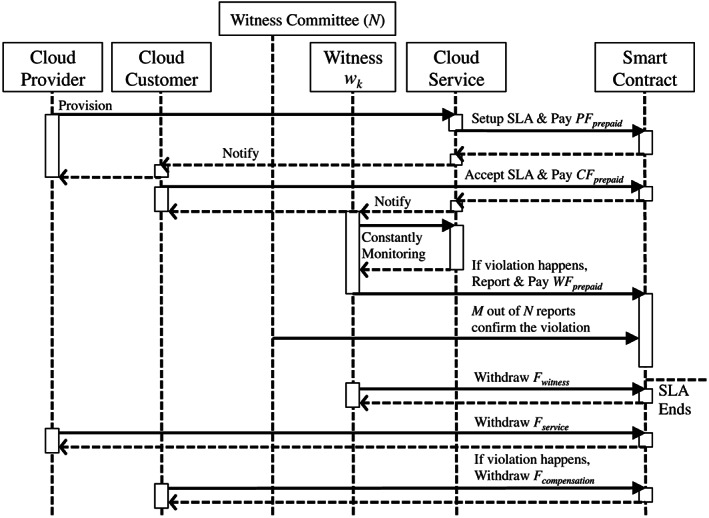
Sequence diagram of different roles in the witness model

The sequence diagram in Figure 2 shows how different roles interact with the smart contract, especially involving the witness in our model. After witnesses being selected, the entire lifecycle of a specific SLA begins. The provider *p* provisions the cloud service and deploy the smart contract on the blockchain. In order to set up an SLA, *p* must prepay the corresponding fee *PF*_*prepaid*_ to the smart contract first. The amount of *PF*_*prepaid*_ is determined by half of the maximum witness fee. The customer *c* is then notified about the service and the content of the smart contract. As all the smart contract on the blockchain is public, the customer can verify the contract and the service status to decide whether to accept the SLA in a particular time window. In order to accept the SLA, the customer also needs to transfer the prepaid fee, *CF*_*prepaid*_. It includes the service fee, *F*_*service*_, and the other half of the maximum witness fee. As we assume *F*_*service*_>*F*_*compensation*_, the compensation fee would be directly deducted from this part of the prepaid fee, if the violation happens. Afterward, every witness in the committee is notified to start monitoring the service continuously.

During the service time, the witness can decide whether to report the event to the smart contract, if there is an SLO violation that for instance, the VM is not accessible. We design the rule that the witness *w*_*k*_ also needs to transfer a small amount of fee, *WF*_*prepaid*_, to endorse its report at the same time. The incentive persuading *w*_*k*_ to report the event is that it would gain relative more rewards as a witness fee if the violation event is finally confirmed by the smart contract. On the contrary, if the violation is not confirmed, *w*_*k*_ would not get back the prepaid endorsement fee, *WF*_*prepaid*_, as a penalty. This design prevents *w*_*k*_ from reporting fake violations just for maximizing its rewards. Moreover, the violation is finally confirmed by the smart contract as the explanation as follows.

Since the first violation report, the smart contract would start counting a time window, *T*_*report*_. Within this time window, the smart contract accepts reports from other witnesses. When the time window *T*_*report*_ is over, the violation is automatically confirmed, if there are no less than *M* out of *N* reports from the witness committee received by the smart contract. *M* is also negotiated by *p* and *c*. It is then defined in the smart contract. Of course, *M* must be bigger than half of *N*. Furthermore, the bigger the *M* is, the more trustworthy the violation is. For example, if there are *N*=3 witnesses in the committee, the SLO violation can only be confirmed when at least *M*=2 of them report the event. Here, it is worth to mention that the smart contract is designed to receive the report only from the committee member. Besides, the second report from the same witness is refused within the same report time window. In some sense, these *N* independent witnesses constitute a *n*−*player* game, in which each witness would like to maximize its rewards. We specially design the payoff function, shown in Section 4.2, and leverage the Nash Equilibrium of game theory to prove that the witness has to be an honest player in this game. That is, they have to report the violation according to the real event.

Finally, the SLA ends in two cases. One case is the service time *T*_*service*_ is over, and there is no violation. The other case is that the SLA is violated. According to these different cases, the three roles can withdraw corresponding fees from the smart contract. Section 4.2 explains more details.

## 4 KEY TECHNIQUES TO ENSURE TRUSTWORTHINESS

In this section, we describe key techniques adopted in our witness model in detail. This model enables the automatic detection on the SLO violation, the results of which can convince both sides: the provider and customer. First, the unbiased random selection algorithm is leveraged to guarantee that most of the witnesses selected into the committee are random and independent. It is also essential to make both sides achieve a consensus that most of the selected witnesses would not delegate the opponent's benefit. Based on this, we give the payoff function for the witness model in Section 3.3. Moreover, through the Nash Equilibrium principle, we prove that the “player” from the witness committee have to behave honestly and tell the truth to maximize its rewards. Furthermore, we analyze some possible fraudulent behaviors from a malicious witness and propose quantitative indications to audit them from the action history.

### 4.1 Unbiased random selection

It is crucial in the witness model that the witness selection for a specific SLA contract is unbiased, ie, neither the provider nor the customer can have advantages in the committee selection. Comparing to our previous work,^25^ we propose a simpler random selection algorithm for committee selection, which is also implemented in the smart contract. We briefly describe it as follows.

(1) There is a basic smart contract to manage the witness pool. It affords a set of interfaces for any blockchain user to register into the pool. Moreover, the registered witness is able to turn its state “Online” or “Offline” in order to indicate whether it can be selected. The detailed witness state management is shown in Section 5.2. A set of interfaces registration allows nodes to register as a witness and to be added in the witness pool. The addresses of the witness pool are managed as a list in the registration order.

(2) The “request” interface is first invoked by a specific SLA contract at block *B*_*b*_. It means this transaction is involved in the block, whose index is *b* ^*th*^. The hash value of this block is $B_{b}^{hash}$. After *K* blocks generated by the blockchain, the other interface “sortition” can be invoked to select *N* online witnesses as candidates. The selection algorithm is shown in Algorithm 1.

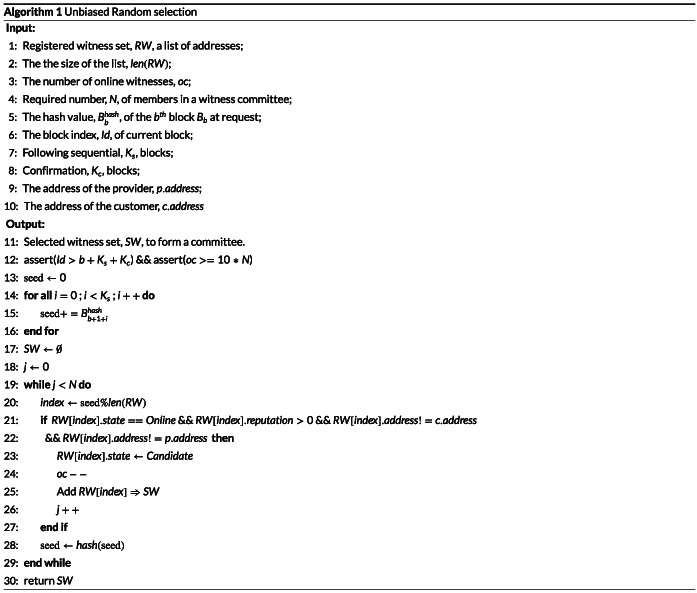

(3) It takes the hash values of the former *K*_*s*_ out of *K* blocks mentioned above as a seed. In addition, we need to wait for other *K*_*c*_ blocks to confirm the adopted recent ones, where the total is *K*=*K*_*s*_+*K*_*c*_. Here, *K*_*s*_ should be chosen such that the probability of some parties sequentially generating *K*_*s*_ blocks are very small. *K*_*c*_ needs to be chosen such that the candidate blocks before are finally involved in the main chain with a dominant probability. These two values depend on the blockchain's own properties. Considering the main net of Ethereum, Gencer et al^26^ showed that the top four miners control 61% of the mining power. Thus, we recommend *K*_*s*_=10 so that with more than 99% probability that the seed is not manipulatable and predictable even if the top four miners collude. On the other hand, it is commonly believed that *K*_*c*_ should be 12. ‖ Considering the average block time of Ethereum is 15 seconds, the duration between the interfaces “request” and “sortition” is less than 6 minutes.

(4) Only the “Online” witness with positive reputation can be selected by the seed from the list of the witness address pool. Anyhow, a new seed is generated based on the hash value of the previous seed. This process is repeated until the required *N* witnesses are selected. At the beginning of Algorithm 1, there are two assertions. We first check whether there has already been the expected number of blocks generated after invoking the “request” interface to ensure the unbiasedness of the random seed from the hash values of these blocks. The other assertion is to make sure that there are at least 10 times of available witnesses than required *N*. It ensures that the number of online witnesses (ie, the potential ones can be selected into the witness committee) is large enough to achieve the randomness among the committee members and prevent collusion. The invoker can only wait for the condition to be met if any of the assertions are violated.

It is worth mentioning that *oc* is leveraged to keep recording how many witnesses are in the “Online” state currently, shown as Figure 4, when the witness is selected by the selection algorithm, and its state turns into “Candidate”, demonstrated as Line 23 of Algorithm 1. The online witness number, *oc*, should be counted down in Line 24.

Considering the difficulty itself of generating a hash value for a block and combining the sequential *K*_*s*_ blocks as seed, we can prove that the selection algorithm is random and unbiased, ie, neither the provider nor the customer can take advantage in the committee with the assumption of trusting the security of Ethereum.

Finally, we analyze the security and trust issues in this design. First, the witness pool should be Sybil‐attack‐proof. This protection can be achieved by requesting a certain amount of deposit to pay to the witness‐pool smart contract when the blockchain participant registers as a witness. Therefore, an entity cannot register a lot of fake witness accounts because that requires a large amount of deposit in total. Second, there is no protection against corruption after the committee candidates are determined. The provider or customer can attack the unwanted candidates to prevent them from joining the committee or bribe them when they are in. However, we argue that this kind of attack or collusion among the provider, customer, and committee members is not easy, ie, (1) the address to identify a witness is the blockchain wallet address in Ethereum, which is just a dynamically user‐generated public key. It is not linked to any real identity or IP address. Therefore, using off‐chain methods, it is even difficult for the witness to convince other committee members that it is also one of the members; (2) any suspicious behavior can be audited, because all the interactions on the blockchain are public and immutable, eg, the possible bribery. Then, the witness' reputation value described in Section 5.2 can be influenced through auditing. Hence, the on‐chain collusion is easy to detect and audit; and (3) when the witness pool is big enough, the unbiased selection algorithm proposed in this section is able to ensure that most of the committee members are not known with each other before and changes every time, since no one can determine the selection result. Therefore, the cost for collusion is high, and the trade‐off prompts the witness to perform honestly for rewards.

### 4.2 Payoff function and nash equilibrium

Game theory targets to mathematically predict and capture behavior in a strategic situation, where each player's rewards depends on the strategies of itself and also others. There is currently a wide range of applications, including economics, evolutionary biology, computer science, political science, philosophy,^27^ and also SLA negotiation.^28^

The strategic or matrix form, of an *n*‐*player* game, is the most common representation of strategic interactions in game theory. The definition consists of a set of players, a set of strategy profiles and a design of payoff functions. Based on the basic type of strategic form game with complete information in game theory, we define our witness game as follows.

Definition 1 Witness Game: It is a *n*‐*player* game represented as a triple (*SW*, Σ, Π), where

- *SW*={*w*_1_,*w*_2_,…,*w*_*n*_} is a set of *n* players. Each player is a selected witness and they form the witness committee.
- Σ=Σ_1_×Σ_2_×…×Σ_*n*_ is a set of strategy profiles, where Σ_*k*_ is a set of actions for the witness *w*_*k*_, ie, *w*_*k*_ can choose any action *σ*_*k*_∈Σ_*k*_. A strategy profile is therefore a vector $\sigma^{*}=(\sigma_{1}^{*},\sigma_{2}^{*},\text{…},\sigma_{n}^{*})$, where $\sigma_{k}^{*}$ is a specific action of Σ_*k*_, (*k*=1,2,..,*n*).
- Π={*π*_1_,*π*_2_,…,*π*_*n*_} is a set of payoff functions, where *π*_*k*_:Σ→*R* is the payoff function determining the rewards for witness *w*_*k*_ under a certain strategy, (*k*=1,2,..,*n*). *R* is the corresponding rewards.

In addition, *σ*_−*k*_={*σ*_1_,*σ*_2_,…,*σ*_*k*−1_,*σ*_*k*+1_,…,*σ*_*n*_} is defined as any strategy profile *σ* without player *k*'s action. The full strategy can then be written as *σ*={*σ*_*k*_,*σ*_−*k*_}. Actually, there are only two actions in our witness game, which is $\Sigma_{k}=\{\sigma_{k}^{\left(r\right)},\sigma_{k}^{\left(s\right)}\}$. $\sigma_{k}^{\left(r\right)}$ means *R*eport the SLO violation to the smart contract. $\sigma_{k}^{\left(s\right)}$ means do not report and keep *S*ilence to the smart contract. In this *N*‐witness game, we define the set of witnesses choosing the action of *R*eport as, *W*_*report*_, where ∀*w*_*k*_∈*W*_*report*_, the $\sigma_{k}^{*}=\sigma_{k}^{\left(r\right)}$. Respectively, *W*_*silence*_ is the set of witnesses not reporting, where ∀*w*_*k*_∈*W*_*silence*_, the $\sigma_{k}^{*}=\sigma_{k}^{\left(s\right)}$. These actions determine the final state of SLA, ie, *SLA*_*status*_=*V*iolated, there is an SLO violation; *SLA*_*status*_=*C*ompleted, service is completed without violation. We then define the violation confirmation as Definition 2.

Definition 2 Violation Confirmation: Based on the result of a strategy profile in a *N*‐witness game, the SLO violation is confirmed, only when ‖*W*_*report*_‖ ≥ *M*, where 1<N/2<M<N,N,M∈N. Otherwise, it is treated as there is no violation happened.

It is worth mentioning that we define *N*>2 and *M*<*N* here, in order to achieve the violation confirmation reliably and fairly. According to our witness model, the witness is designed to report the violation along with endorsement fee to the SLA smart contract. Therefore, if the violation is not confirmed, the witness cannot retrieve back its endorsement fee as a penalty. The detailed payoff function design is shown as Definition 3according to the aforementioned definitions and analysis. Thereinto, the value of each function is only leveraged to quantitatively represent the relative relationship among the fees. Hence, 1 represents one share of profit. 10 is ten times shares of 1. −1 means losing one share of profit.

Definition 3 Payoff Functions: The values of payoff functions are designed according to the final SLA status.

• When *SLA*_*status*_=*V*iolated:                                                                                   • When *SLA*_*status*_=*C*ompleted:
 
$\forall w_{k}\in W_{report},\pi_{k}(\sigma_{k}^{\left(r\right)},\sigma_{-k})=10$; $\forall w_{k}\in W_{report},\pi_{k}(\sigma_{k}^{\left(r\right)},\sigma_{-k})=-1$;
 
$\forall w_{k}\in W_{silence},\pi_{k}(\sigma_{k}^{\left(s\right)},\sigma_{-k})=0$. $\forall w_{k}\in W_{silence},\pi_{k}(\sigma_{k}^{\left(s\right)},\sigma_{-k})=1.$

In a *n*‐*player* game, if a player knows the others' actions, it would choose a strategy to maximize its payoff. This is referred as its best response. Therefore, the best response of the witness *w*_*k*_ can be defined as follows.

Definition 4 Witness $w_{k}$'s best response: In order to maximize its rewards, $w_{k}$'s best response to a strategy profile $\sigma_{-k}^{*}$ is a strategy $\sigma_{k}^{*}\in \Sigma_{k}$, such that $\pi_{k}(\sigma_{k}^{*},\sigma_{-k}^{*})\geq \pi_{k}(\sigma_{k},\sigma_{-k}^{*})$ for ∀*σ*_*k*_∈Σ_*k*_(*k*=1,2,…,*n*).

A Nash Equilibrium point^27^ is therefore able to be defined as a stable state, where no player has an incentive to deviate from current strategy. It is actually a strategy under which every player adopts its own best response.

Definition 5 Nash Equilibrium point: It is a specific strategy profile $\sigma^{*}=(\sigma_{k}^{*},\sigma_{-k}^{*})$, if for every witness *w*_*k*_, $\sigma_{k}^{*}$ is a best response to $\sigma_{-k}^{*}$, ie, ∀*w*_*k*_∈*SW* and ∀*σ*_*k*_∈Σ_*k*_(*k*=1,2,…,*n*), $\pi_{k}(\sigma_{k}^{*},\sigma_{-k}^{*})\geq \pi_{k}(\sigma_{k},\sigma_{-k}^{*})$.

Based on the Nash Equilibrium point definition and payoff functions, we can derive the theorem as follows.

Theorem 1 In a witness game, the only two Nash Equilibrium points are following strategy profiles:

- $\sigma^{*}=(\sigma_{1}^{*},\sigma_{2}^{*},\text{…},\sigma_{n}^{*})$, of which ∀*w*_*k*_∈*SW*, $\sigma_{k}^{*}=\sigma_{k}^{\left(r\right)}$;
- $\sigma^{*}=(\sigma_{1}^{*},\sigma_{2}^{*},\text{…},\sigma_{n}^{*})$, of which ∀*w*_*k*_∈*SW*, $\sigma_{k}^{*}=\sigma_{k}^{\left(s\right)}$.

According to Definitions 1 and 2, in a *N*‐witness game, *N* ≥ 3, *N*/2<*M* ≤ *N*−1 and *M* ≥ 2, where N,M∈N.

For the strategy profile of ∀*w*_*k*_∈*SW*, $\sigma_{k}^{*}=\sigma_{k}^{\left(r\right)}$, which means ‖*W*_*report*_‖=*N*>*M*. The SLO violation status is therefore violated, *SLA*_*status*_=*V*. According to payoff functions design in Definition 3, for ∀*w*_*k*_, its rewards is $\pi_{k}(\sigma_{k}^{\left(r\right)},\sigma_{-k}^{*})=10$. If any *w*_*k*_ chooses the other action, *S*ilence, instead of *R*eport. The final status of SLA, however, would not be modified, due to ‖*W*_*report*_‖=*N*−1 ≥ *M*. Then, $w_{k}^{\prime }$s rewards is $\pi_{k}(\sigma_{k}^{\left(s\right)},\sigma_{-k}^{*})=0<10=\pi_{k}(\sigma_{k}^{\left(r\right)},\sigma_{-k}^{*})$. According to Definition 5, this strategy profile is a Nash Equilibrium point.

Analogously, for the strategy profile of ∀*w*_*k*_∈*SW*, $\sigma_{k}^{*}=\sigma_{k}^{\left(s\right)}$, which means ‖*W*_*report*_‖=0<2 ≤ *M*. The SLO violation status is therefore not violated, *SLA*_*status*_=*C*. According to payoff functions design in Definition 3, for ∀*w*_*k*_, its rewards is $\pi_{k}(\sigma_{k}^{\left(s\right)},\sigma_{-k}^{*})=1$. If any *w*_*k*_ chooses the other action, *R*eport, instead of *S*ilence. The final status of SLA, however, would not be modified, due to ‖*W*_*report*_‖=1<2 ≤ *M*. Then, *w*_*k*_'s rewards is $\pi_{k}(\sigma_{k}^{\left(r\right)},\sigma_{-k}^{*})=-1<1=\pi_{k}(\sigma_{k}^{\left(s\right)},\sigma_{-k}^{*})$. According to Definition 5, this strategy profile is also a Nash Equilibrium point.

For all the other strategy profiles, they are all a mix of actions, *R*eport and *S*ilence. It means *W*_*report*_≠*∅* and *W*_*silence*_≠*∅*. When *SLA*_*status*_=*V*, ie, ‖*W*_*report*_‖ ≥ *M*, there is always ∃*w*_*k*_∈*W*_*silence*_; it can change the action to *R*eport. However, the SLA status would not change, because of ‖*W*_*report*_‖+1>*M*. Hence, *w*_*k*_ increases its rewards, from $\pi_{k}(\sigma_{k}^{\left(s\right)},\sigma_{-k}^{*})=0$ to $\pi_{k}(\sigma_{k}^{\left(r\right)},\sigma_{-k}^{*})=10$. On the other hand, when *SLA*_*status*_=*C*, ie, ‖*W*_*report*_‖<*M*, there is always ∃*w*_*k*_∈*W*_*report*_; it can change the action to *S*ilence. However, the SLA status would not change, because of ‖*W*_*report*_‖−1<*M*. Hence, *w*_*k*_ increases its rewards, from $\pi_{k}(\sigma_{k}^{\left(r\right)},\sigma_{-k}^{*})=-1$ to $\pi_{k}(\sigma_{k}^{\left(s\right)},\sigma_{-k}^{*})=1$. These counterexamples demonstrate all these strategy profiles are not Nash Equilibrium points.

Therefore, in a witness game, there are two and only two Nash Equilibrium points. They are $\sigma^{*}=(\sigma_{1}^{\left(r\right)},\sigma_{2}^{\left(r\right)},\text{…},\sigma_{n}^{\left(r\right)})$ and $\sigma^{*}=(\sigma_{1}^{\left(s\right)},\sigma_{2}^{\left(s\right)},\text{…},\sigma_{n}^{\left(s\right)})$.  

We take the basic *three*‐witness game as an example, where *N*=3. Therefore, *M* can only be equal to 2 based on Definition 2. Table 1 shows payoff functions according to our previous definitions. The value element in Table 1 is the vector of corresponding payoff function values. It is represented as (*π*_1_,*π*_2_,*π*_3_). According to Theorem 1, Nash Equilibrium points in this game are (10, 10, 10) and (1, 1, 1), respectively.

**TABLE 1:** Payoff functions for a *three*‐witness game

	***w***_3_			
	$\sigma_{3}^{\left(r\right)}$: *R*eport		$\sigma_{3}^{\left(s\right)}$: *S*ilence	
***w***_1_	***w***_2_		***w***_2_	
	$\sigma_{2}^{\left(r\right)}$: *R*eport	$\sigma_{2}^{\left(s\right)}$: *S*ilence	$\sigma_{2}^{\left(r\right)}$: *R*eport	$\sigma_{2}^{\left(s\right)}$: *S*ilence
$\sigma_{1}^{\left(r\right)}$: *R*eport	(10, 10, 10)	(10, 0, 10)	(10, 10, 0)	(‐1, 1, 1)
$\sigma_{1}^{\left(s\right)}$: *S*ilence	(0, 10, 10)	(1, 1, ‐1)	(1, ‐1, 1)	(1, 1, 1)

Based on the aforementioned analysis, for a rational and selfish witness, who wants to maximize its rewards through offering services, would have to behave as follows in this game. If there is a violation happening, the witness knows that most of other witnesses are more likely to report this event to gain more rewards. Hence, the higher rewards push the witness to report this event. On the contrary, if there is no violation, the witness knows that most of other witnesses are more likely to keep silence. Although the witness wants to achieve the highest rewards, it has to take a great risk to pay a penalty for its fraudulent behavior. From the global view, when there is no violation, all the witnesses prefer to keep silence in order to stay at the Nash Equilibrium point, $\left(\sigma_{1}^{\left(s\right)},\sigma_{2}^{\left(s\right)},\text{…},\sigma_{n}^{\left(s\right)}\right)$. Then, the violation acts as a signal to push them achieving another Nash Equilibrium point, $\left(\sigma_{1}^{\left(r\right)},\sigma_{2}^{\left(r\right)},\text{…},\sigma_{n}^{\left(r\right)}\right)$, with much higher rewards. At the same time, they tell the truth about the SLO violation.

Therefore, it is not the witness who wants to tell the truth. Instead, it has to be honest, in order to maximize its rewards.

### 4.3 Witness audit

The selection algorithm, Algorithm 1, ensures that the selected witnesses are independent to a great extent. The payoff function design stimulates the witness telling the truth. However, an auditing mechanism is still needed to ensure that the malicious or irrational witness can be detected and kicked out from the witness pool. As all the interactions with the smart contract, ie, transactions, are public and permanently stored on the blockchain, it is possible to audit a witness through its behavior history. We mainly exploit the following information in the history to do auditing. It is expressed as Equation . It represents a set of events of the witness, *w*_*k*_. The witness can adopt the action of silence $\sigma_{k}^{\left(s\right)}$ or the action of report $\sigma_{k}^{\left(r\right)}$. $\sigma_{k}^{*}$ means the SLA event with any action adopted by *w*_*k*_. To be specific, when the action is report, we can also further know its *order* among all the witnesses' reports, first to report or not, and the reporting time *T*_*r*_, relatively from the SLA starting time. In addition, *status* expresses whether the SLA is violated or completed. ∗ means the event with any SLA status is counted in this set

(1) $$Event\left(w_{k},\sigma_{k}^{\left(s\right)}\left|\left[\sigma_{k}^{\left(r\right)}:(order,T_{r})\right]\right|\sigma_{k}^{*},status:C|V|*\right).$$

Based on the information above, we analyze that there are three types of malicious witnesses, ie, lazy witness, speculative witness, and sacrificed witness.

***Lazy witness*** refers to the one, who prefers not to report the violation. Since there is a case that the higher incentive for reporting a violation is not enough to motivate the lazy one, they can choose the strategy not to really monitor the service. Then, they always keep silence and never report the violation. With this strategy, they would not pay the penalty, even if the final status of SLA is actually violated. Considering violation is not a regular event, the lazy witness is still able to gain some rewards via multiple “games”. However, this type of lazy witness can be audited through the active rate, which is defined as follows.

Definition 6 Active rate (*η*_*active*_(*w*_*k*_)): This is the metric to measure the activeness of witness *w*_*k*_, when there is a violation

(2) $$\eta_{active}(w_{k})=\frac{\left\|Event(w_{k},\sigma_{k}^{\left(r\right)},status:V)\right\|}{\left\|Event(w_{k},\sigma_{k}^{*},status:V)\right\|}.$$

*η*_*active*_(*w*_*k*_)=0 means that the witness *w*_*k*_ never reports the violation event, although there actually is one. A threshold, ${\hat{\eta }}_{active}(w_{k})$ therefore can be set to determine whether *w*_*k*_ is a lazy witness, ie, still $\eta_{active}(w_{k})<{\hat{\eta }}_{active}(w_{k})$, when *w*_*k*_ has already been involved into many SLA events.

***Speculative witness*** refers to the one, who is more likely to report in a speculative way. Since all the actions are public and transparent on the blockchain, there is a possible speculative behavior for the witness, which is only to follow others' report. The witness does not monitor the service. Instead, it monitors transactions on the blockchain to see whether some other witness is reporting the violation. Then, it immediately follows and reports, trying to gain the maximum rewards. Although we can set a relatively short report time window in the model design of Section 3.3, its speculative reports might still be counted in the following blocks of the blockchain. Moreover, this speculative witness also needs to take the risk that the violation may not be confirmed finally. Anyhow, this type of speculative witness can be audited through the following rate.

Definition 7 Following rate (*η*_*follow*_(*w*_*k*_)): This is the metric to measure the frequency that the witness *w*_*k*_ follows the reports of other witnesses

(3) $$\eta_{follow}(w_{k})=\frac{\left\|Event\left(w_{k},\sigma_{k}^{\left(r\right)}:(NotFirst,T_{r}),status:V\right)\right\|}{\left\|Event\left(w_{k},\sigma_{k}^{\left(r\right)}:(order,T_{r}),status:V\right)\right\|}.$$

Here, *NotFirst* means that the transaction containing the report of *w*_*k*_ is not the first block in the reporting time window. Therefore, *η*_*follow*_(*w*_*k*_)=100*%* means for all violated SLA events involving the witness *w*_*k*_, it is never the first one to report. A threshold, ${\hat{\eta }}_{follow}(w_{k})$ therefore can be set to determine whether *w*_*k*_ is a speculative witness through following, ie, when $\eta_{follow}(w_{k})>{\hat{\eta }}_{follow}(w_{k})$, if *w*_*k*_ is involved into many SLA events.

***Sacrificed witness*** refers to the one, who always reports at a specific time stamp. For instance, *w*_*k*_ always reports the violation within one minute after the SLA starts. Although the witness may pay a lot of penalty for its malicious behavior at the beginning, it can show other witnesses its behavior pattern from its history later on. In some sense, it is able to imply to others that it would report at some time stamp. Then, as long as others have analyzed its behavior pattern and followed, it can most likely gain the maximum rewards. Hence, it is crucial to audit this type of witness through the following fixed pattern rate.

Definition 8 Fixed pattern rate (*η*_*fix*_(*w*_*k*_)): This is the metric to measure the frequency that the witness *w*_*k*_ reports the violation at a specific time stamp, ${\hat{T}}_{r}$

(4) $$\eta_{fix}(w_{k})=\frac{\left\|Event\left(w_{k},\sigma_{k}^{\left(r\right)}:(order,{\hat{T}}_{r}),status:*\right)\right\|}{\left\|Event(w_{k},\sigma_{k}^{*},status:*)\right\|}.$$

Here, *η*_*fix*_(*w*_*k*_)=100*%* means for all of the SLA events, which involves the witness *w*_*k*_, it always reports at time stamp, ${\hat{T}}_{r}$. A threshold, ${\hat{\eta }}_{fix}(w_{k})$ therefore can be set to determine whether *w*_*k*_ is a sacrificed witness through following, ie, when $\eta_{fix}(w_{k})>{\hat{\eta }}_{fix}(w_{k})$, even if *w*_*k*_ is just involved into several SLA events.

It is worth to mention that these auditing mechanisms can also be implemented in the smart contract, in order to avoid a third party to dominate the judgment. It can be combined with the reputation value of the witness, which is further explained as the witness reputation in the implementation part of Section 5.2. Therefore, when the witness' reputation decreases to zero, that witness would also be blocked automatically by the selection algorithm.

## 5 PROTOTYPE IMPLEMENTATION AND EXPERIMENTS

According to the witness model and the payoff function design, we implement a prototype system based on the smart contracts of Ethereum. We leverage the programming language, Solidity, ** provided by Ethereum, to program smart contracts. In this section, we first would like to discuss the overall relationship among different roles and smart contracts in our SLA system. Afterward, we illustrate the state transition in the smart contract of witness pool and SLA, respectively. Via this, we describe the detailed functionalities of the interfaces in the smart contracts and show how they are leveraged to transit the states. Finally, we show some experimental study on the transaction cost of these interfaces on the Ethereum test net, “Rinkeby”.

### 5.1 Overall system implementation

**FIGURE 3:**
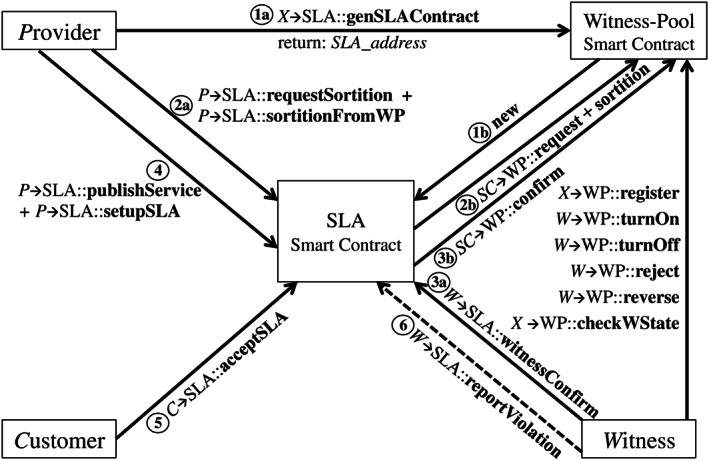
Interactions among roles and smart contracts

**FIGURE 4:**
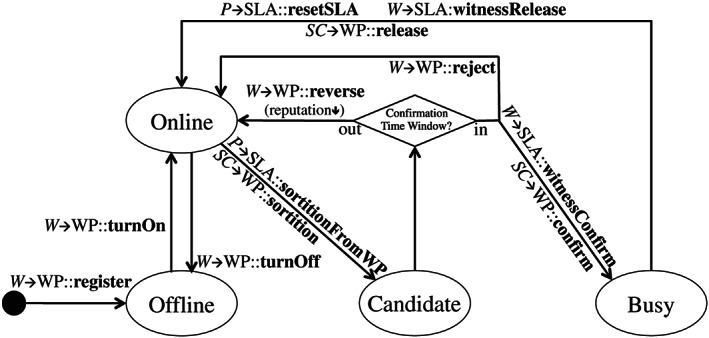
Witness state transition diagram in the witness pool smart contract

**FIGURE 5:**
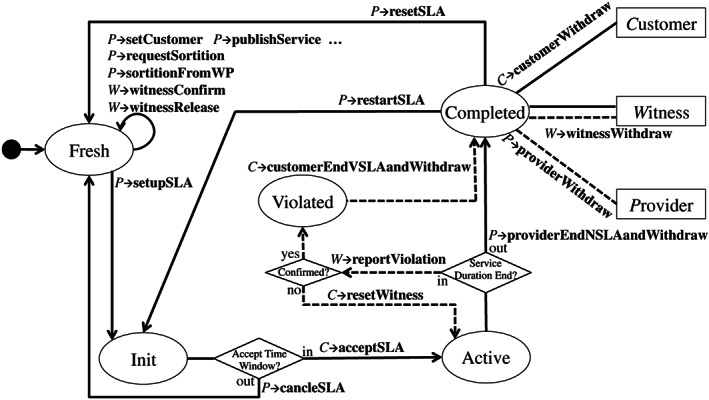
State transition diagram of SLA lifecycle for a specific SLA smart contract

Overall, there are three roles and two types of smart contracts in our SLA enforcement system. Roles include the traditional *P*rovider and *C*ustomer, as well as the introduced *W*itness. The smart contracts include the witness‐pool smart contract and the SLA smart contract. Figure 3 illustrates the relationship among these entities through different interfaces. These interfaces are named as the text on the arrow. The format of the text is “*R*_*role*_→ [*C*_*type*_::]*N*_*interface*_”. It means that only the role *R*_*role*_ can invoke the interface, *N*_*interface*_, which is defined in the smart contract of *C*_*type*_. The corresponding implementation is achieved by the checking mechanism, which is the property of the programming language provided by Ethereum. Therefore, the smart contract restricts that only the specified role can interact with the smart contract in a certain state. The representation of the *R*_*role*_ are *P* for *P*rovider, *C* for *C*ustomer, *SC* for a generated SLA *S*mart *C*ontract, and *X* for any blockchain user. Moreover, for *C*_*type*_, *WP* is for the witness‐pool type of smart contract, and *SLA* is for the generated ones for SLA enforcement. In addition, this interface definition also applies to Figures 4 and 5.

The witness‐pool smart contract is the basis in order to set up the system. Any blockchain user can register and become the *W*itness role through the interface “register” provided by the witness‐pool smart contract. There are also some other interfaces for the witness to invoke to change its state in the pool in order to be selected. More details are discussed in Section 5.2. For a specific SLA lifecycle, any user *X* can invoke the interface “genSLAContract” provided by the witness‐pool smart contract. Afterward, a specific SLA smart contract is generated by the witness‐pool smart contract, and the contract address is returned back. This address is also recorded in the witness‐pool. It ensures the validity of the SLA smart contract to interact with other roles. Meanwhile, the user *X* becomes the *P*rovider role of the generated SLA smart contract. It can customize the contract, including setting the customer's address, and other negotiated contract parameters, such as service duration and witness committee scale *N*. It is also responsible for performing the unbiased random selection algorithm, explained in Section 4.1. As this selection algorithm is leveraged by the provider through a specific SLA contract, the online witness can know it is selected by which SLA smart contract, basically the contract address, from checking its own state. Then, it can make a confirmation to the SLA contract to join the witness committee. Simultaneously, the SLA smart contract further invokes interfaces of witness‐pool smart contract to acknowledge the witness management. After a proper witness committee is constructed, the provider can publish its service detail on the chain to initiate the SLA lifecycle. Details are explained in Section 5.3.

### 5.2 Witness‐pool smart contract implementation

In this part, we focus on implementation details about the witness‐pool smart contract, especially the witness management. Figure 4 illustrates the states of a witness role defined in the smart contract. It includes four states, ie, “Online”, “Offline”, “Candidate”, and “Busy”. The state transition of the witness is as follows.

After registration in the witness pool, only the witness itself can turn its state into “Online”. It then probably selected by a specific SLA smart contract. Hence, it needs to monitor its own state on the blockchain continuously. This operation is feasible, as the read‐only operation does not need any transaction fee. Once it is selected by a specific smart contract through performing “sortition” algorithm, its state turns into “Candidate”. Within a confirmation time window, eg, 2 minutes, the witness can look through the SLA smart contract, which selects it, and decide whether to confirm or reject this selection. If it rejects, the provider of the SLA smart contract has to perform another selection. Otherwise, its state turns into “Busy”, after it invokes the interface, “witnessConfirm”, of the SLA smart contract. By the end of each SLA lifecycle iteration, the witness has the right to actively leave the SLA contract by leveraging the interface “witnessRelease”. On the other hand, it can also be passively released from the SLA contract if the provider invokes the interface “resetSLA” to dismiss the witness committee. Finally, the witness can “trunOff” to avoid being selected before it is not available to the Internet.

In order to prevent some malicious intentions, we bring in a reputation value for each witness to measure their behaviors. First, each witness has an initial reputation value of *R*_*init*_ at registration, which could be a predetermined constant value. Then, for instance, some witness may not turn its state into “Offline”, when it is not actually available or does not frequently check its state. Then, it would not be able to confirm the selection and join the SLA contract within the confirmation time window, if it is chosen. In this case, the witness would not be chosen again, since its state becomes “Candidate”. To reverse back to the state “Online”, in which it can be selected, the witness has to leverage the interface, “reverse”. In this case, its reputation value decreases by 10. If this value becomes zero or less, it would be permanently blocked by the selection process according to Algorithm 1. We also combine the reputation value with the auditing mechanism mentioned above in some sense. For example, the reputation of the witness, who does not report, would decrease by 1, when the violation is confirmed. It is the same with the one, who reports the violation but not finally confirmed.

It is worth mentioning that in the current implementation, we have not designed the scenario where the reputation can be heightened. The idea is to make the reputation decreasing as a soft punishment, not directly losing tokens (money). However, when the reputation is too low, the account is blocked, and the witness has to register another account. Meanwhile, the witness would lose the deposit of the blocked account in this case. On the hand, if the witness can heighten its reputation through performing honestly, even the increasing scale is much smaller than the decreasing scale. This design would still give witnesses the chance to balance the reputation through performing different times of honest behaviors and malicious behaviors, eg, performing maliciously once and ten times of honest behaviors afterward.

### 5.3 SLA smart contract implementation

Figure 5 shows the SLA state transition to implement a specific SLA smart contract enforcement. This type of smart contract is generated by the witness‐pool smart contract. All the interfaces annotated in this figure belongs to this type of SLA smart contract. Hence, we omit the definition scope *C*_*type*_::. There are five states, ie, “Fresh”, “Init”, “Active”, “Violated”, and “Completed”, shown as circle in Figure 5. The dashed arrows demonstrate the state transition path when a violation happens. The three squares in the figure represent the respective roles in this smart contract. At the end of SLA, they can withdraw the rewards, respectively. The dashed line here also refers to the action adopted under the situation of the violation.

The contract is generated in the state of “Fresh”. In this state, the provider can customize the SLA parameters according to the negotiated results with the customer. Furthermore, the service detail can also be published onto the contract through “publishService”. In our case, the detail is the public IP of the VM. All others are therefore being notified. However, this SLA only proceeds when the number of the members in the witness committee is satisfied. Otherwise, the provider is unable to leverage the interface, “setupSLA”, to transit into “Init” state. According to the witness model design in Section 3.3, the provider needs to prepay some fee, *PF*_*prepaid*_, to the smart contract for hiring witnesses. The actual amount of fee is calculated by the smart contract according to the scale of committee member and a basic hiring fee. Moreover, this amount of tokens is one of the requirements to invoke the interface. It ensures that only that amount of prepaid fee is transferred into the smart contract. The customer then decides whether to accept the SLA. If it accepts the SLA, it also needs to prepay the fee, *CF*_*prepaid*_, including the service fee and its part of the hiring fee for witnesses. If not, the provider can “cancelSLA” and withdraw back its money. When the service is completed, all corresponding roles can retrieve their rewards through a set of withdrawing interfaces. After all the money is withdrawn from the contract, the provider can leverage “resetSLA” or “restartSLA” to rotate back to the previous state. These interfaces are designed for continuous service delivery instead of a long service duration to stuck witnesses. The difference between these two interfaces is “resetSLA” dismisses the witness committee and the provider can change some other terms. On the other hand, “restartSLA” would keep the committee and quickly proceed another round of SLA iteration.

### 5.4 Experimental study

In order to test all the functionalities of our model and system design, we deploy the implemented smart contracts on the test net of Ethereum blockchain, “Rinkeby”. It is a world‐wide test blockchain for developers to debug the smart contract. The “Ether”, which is the cryptocurrency of Ethereum, does not worth real value on the test net and can be applied for debugging. Hence, we generate several accounts on “Rinkeby” to simulate different roles, ie, the provider, customer, and witnesses. We leverage the retrieved “Ether” on each simulated account to execute the interfaces and prepay different types of fees according to the model. To conduct the experiment, we first deploy the basic witness‐pool smart contract and make all the accounts registered to the witness pool. The provider then generates an SLA smart contract to start the SLA lifecycle with the customer. Afterward, we test all possible scenarios to exploit and validate the functionality of different interfaces. The results demonstrate that our system implementation satisfies our model and payoff function design.

The trust part of the system is proved by game theory and ensured by the unbiased selection algorithm, whose credibility is endorsed by the blockchain technique. Therefore, we mainly analyze some performance information from our experimental study. Here, the performance refers to the complexity of each interface in the smart contract. It determines the transaction fee needed to pay the miner in Ethereum since the miner needs to execute the program defined in the interface, which consumes the electricity power of the miner. The more complex of the interface is the more transaction fee required when it is invoked. The transaction fee is measured as “Gas” defined in Ethereum, which is a unit that refers to how much work taken by the miner when executing the transaction. The final fee is the product of gas amount and the gas price for each unit. Hence, the gas consumption is similar no matter on the test net or main net. We therefore record all the gas consumption for each interface from the transaction history of the experiment.

**FIGURE 6:**
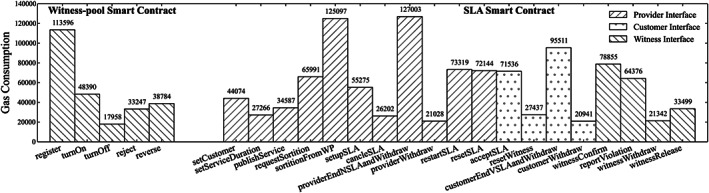
The gas consumption for each interface in smart contracts

Figure 6 illustrates the gas consumption of each interface. We show the major interfaces defined in the two types of smart contracts, which construct the system. Some simple interfaces, such as the one that sets the SLA parameters, are omitted. Their gas consumptions are similar to “setServiceDuration” in the figure. Some other interfaces of the witness‐pool smart contract are also omitted, as these are the ones that can only be invoked by the SLA smart contract. Its consumption is involved in some interface in the right part of the figure. Besides, the consumption of “genSLAContract” in the witness‐pool smart contract is also not shown in the figure, because it is more than 10 times higher than others, which is around 2 200 958. However, it is acceptable for the provider to invoke this to generate a new SLA smart contract, especially the SLA smart contract can be reusable.

From the experimental study, it can be derived that, compared with the customer and the witness, the provider tends to require more gas in the entire SLA lifecycle. The interfaces of customer and witness consume less. The consumption of different roles fits our model design and reality, because in most cases, the provider earns the most rewards through offering service. It has the incentive to proceed with the lifecycle. The lightweight gas consumption for witness role is also able to convince blockchain users to take part in the system to work as a witness. Moreover, these gas consumption values are achieved through experiments based on the current implementation. There is still possible space to optimize the interface implementation further, in order to lower the gas consumption.

## 6 CONCLUSION

In this paper, a witness model has been proposed for cloud SLA enforcement and specially design the payoff function for each witness. We leverage the game theory to analysis that the witness has to offer honest monitoring service in order to maximize its own rewards. Finally, a prototype system is fully implemented using smart contracts of Ethereum to realize the witness model, not only the SLA enforcement lifecycle but also the witness management of witness pool is implemented with the smart contract. The experimental study demonstrates the feasibility of our model and shows the system performance. Via this way, the trust problem is transferred to economic issues. It is not the witness itself would like to be honest, but the economic principles force them to tell the truth. Here, the blockchain plays as public and immutable monetary management platform according to some predefined rules. We also believe the witness model can be applied in other scenarios with blockchain, where originally only two roles are involved in a contract, and oracles are required to provide results of off‐chain events.

For future work, there are mainly two directions, ie, on‐chain and off‐chain. For the on‐chain part of work, we are going to further optimize the interface implementation to reduce the gas consumption and enrich the functionalities of the smart contract. Besides, some more scenarios should be considered to apply our model. Moreover, a more practical mechanism for the witnesses to achieve consensus on the off‐chain events should be considered. For the off‐chain part of work, user‐friendly tools are going to be developed for each role in the system to monitor the state on the chain and perform their corresponding interactions. On the other hand, it can also be combined with our cloud application DevOps framework, CloudsStorm,^29^ to construct the witness ecosystem. The vision is to ensure cloud performance for applications through automated SLA.

## Footnotes

* https://aws.amazon.com/compute/sla/

† https://blog.ethereum.org/2014/07/22/ethereum‐and‐oracles/

‡ http://www.oraclize.it

§ https://github.com/zh9314/SmartContract4SLA

¶ https://www.rinkeby.io/

# http://aws.amazon.com/cloudwatch/

‖ https://blog.ethereum.org/2015/09/14/on‐slow‐and‐fast‐block‐times/

** http://solidity.readthedocs.io
